# Monitoring Gases Content in Modern Agriculture: A Density Functional Theory Study of the Adsorption Behavior and Sensing Properties of CO_2_ on MoS_2_ Doped GeSe Monolayer

**DOI:** 10.3390/s22103860

**Published:** 2022-05-19

**Authors:** Xin Gao, Yunwu Li

**Affiliations:** 1 College of Engineering and Technology, Southwest University, Chongqing 400716, China; swugaoxinxi@163.com; 2 School of Computer Science & Technology, Beijing Institute of Technology, Beijing 100081, China

**Keywords:** gas sensors, density functional theory, 2D materials, carbon dioxide

## Abstract

The reasonable allocation and control of CO_2_ concentration in a greenhouse are very important for the optimal growth of crops. In this study, based on density functional theory (DFT), an MoS_2_–GeSe monolayer was proposed to unravel the issues of the lower selectivity, poorer sensitivity and non-recyclability of traditional nanomaterial gas sensors. The incorporation of MoS_2_ units greatly enhanced the sensitivity of the pure GeSe monolayer to CO_2_ and the high binding energy also demonstrated the thermal stability of the doped structures. The ideal adsorption energy, charge transfer and recovery time ensured that the MoS_2_–GeSe monolayer had a good adsorption and desorption ability. This paper aimed to solve the matter of recycling sensors within agriculture. This research could provide the theoretical basis for the establishment of a potentially new generation of gas sensors for the monitoring of crop growth.

## 1. Introduction

In modern agriculture, greenhouses are often used to artificially change the growth environment of crops in order to cope with random and changeable weather conditions and ensure the diversity of seasonal agricultural products [1,2,3]. In the growth process of plants, carbon is an important organic matter, mainly in the form of CO_2_ [4,5]. CO_2_ is a necessary material for plants, which plays a very important role in photosynthesis [6,7,8]. It directly affects the normal growth of plants and the abundance of nutrients within plants. In addition, the concentration of CO_2_ can change the biological and abiotic conditions in soil to some extent [9,10]. Increases in the emissions of potent greenhouse gases from soil under increasing atmospheric CO_2_ indirectly affect the growth of crops. Moreover, the demand for CO_2_ is different in different growth stages of crops. For example, in this study, demand for CO_2_ reached the maximum value of 1000–1200 ppm before sunrise and then decreased to about 100 ppm 2.5–3 h after sunrise, which was only about 30% of the atmospheric concentration. It did not begin to rise again until 2 h after sunrise and returned to the atmospheric level around 4 p.m. The concentration of carbon dioxide that is required for vegetables to grow is generally 1000~1500 ppm, so CO_2_ deficiency in a greenhouse is quite serious and has become an important factor that affects the yield of greenhouse vegetables. Therefore, the reasonable allocation and control of CO_2_ concentration in greenhouses are critical for the optimal growth of crops. Zhang et al. have conducted a lot of research on the study of density functional theory (DFT) [11,12,13,14,15,16]. They studied the microscopic interactions between two-dimensional (2D) materials and gas molecules to explore the application of these materials as gas adsorbents and sensors, such as the use of an Au-doped graphene monolayer as a gas sensor [13,16]. Other relevant work has proved that the simulation calculation is very consistent with experimental results. Based on DFT, we theoretically designed a new type of CO_2_ concentration detection sensor, which can be indirectly observed and used to control the growth of plants.

Compared to traditional gas sensors, the use of nanomaterial sensors for the detection of gas type and gas content has the advantages of small size, fast response, high sensitivity and low costs. Tao et al. explored the use of nanomaterial sensors, both in theory and experimentally [17,18,19,20,21]. Thanks to their large surface areas and adequate adsorption sites, 2D materials are increasingly being used as gas adsorbents and sensors and were studied by Gui et al. [22,23,24,25]. After graphene, GeSe monolayers have attracted a lot of interest as 2D materials with high optical responsiveness, high mobility and high flexibility. Zhou et al. also systematically explored the physical and chemical properties of a GeSe monolayer [26,27,28] and Dai et al. successfully prepared GeSe thin films [29]. Recently, several researchers have explored the use of GeSe monolayers as gas sensors or adsorbents. Liu et al. studied the gas adsorption characteristics of an intrinsic GeSe monolayer [30], but due to its weak adsorption ability, it could not provide enough of a theoretical basis for gas sensors. Tao et al. explored the adsorption characteristics of a modified GeSe monolayer for SF_6_ decomposition gas by doping metal oxides [31,32,33]. However, despite being an important gas that is applied in various fields, CO_2_ lacks theoretical exploration using GeSe-based monolayers. Li et al. found that the adsorption ability of Ce–CaO to CO_2_ is too strong to be desorbed, which is not conducive to the recyclability of sensors [34,35,36,37]. The adsorption ability of Al–BN monolayers and graphene to CO_2_ is also too small, which is the reason that they cannot obtain enough of a response [38,39]. Gui et al. proposed that metal oxide doping can effectively improve the gas selectivity and sensitivity of 2D materials [40,41], which offered the essential theoretical basis for our study.

Here, we innovatively propose the use of a MoS_2_–GeSe monolayer to detect CO_2_ for the first time. Based on the first principle, parameters such as binding energy (*E*_b_), adsorption energy (*E*_ads_), charge transfer (Δ*Q*), band gap (*E*_g_), density of states (DOS), work functions (WFs), charge density difference (CDD), deformation charge density (DCD) and electron localization function (ELF) were analyzed. Doping with MoS_2_ units caused the GeSe monolayer to have a higher sensitivity to CO_2_ and an excellent thermal stability (*E*_b_ = −5.994 eV). For the better simulation of the practical applications of gas sensors, we analyzed the recovery time at different temperatures. Appropriate *E*_ads_ (−0.955 eV) and Δ*Q* (0.266 e) values caused the MoS_2_–GeSe monolayer to maintain an excellent response and also produce great recovery characteristics (1.25 s in 398 K). This could provide solid theoretical support for the application of resistive chemical sensors in agriculture.

## 2. Methods

All calculations were performed using the DMOL^3^ and CASTEP frameworks [42,43]. The generalized gradient approximation (GGA) and the Perdew–Burke–Ernzerhof (PBE) exchange correlation function were selected for analysis [44]. The energy convergence accuracy, maximum force and maximum displacement were set as 1 × 10 ^−5^ Ha, 2 × 10 ^−3^ Ha/Å and 5 × 10 ^−3^ Ha/Å, respectively. Considering the weak interaction between atoms in calculations, such as the van der Waals force, Grimme’s DFT-D was applied [45,46,47,48]. Double numerical polarization (DNP) was also selected at the basic setting of 3.5. 

A 4 × 4 × 1 GeSe monolayer supercell was constructed and optimized using the highest precision setting in the material studio software. The k-point samples of the Monkhorst–Pack grid were sampled at 6 × 6 × 1 for geometry optimization and 10 × 10 × 1 for electronic optimization. The size of the vacuum space between two adjacent sheets was set at the value of 15 Å in order to avoid interactions between periodic images. A self-consistent loop energy of 10^−6^ Ha, a global orbital cut-off radius of 5.0 Å and a smearing of 0.005 Ha were applied. In order to study the role of MoS_2_ doping in the adsorption of CO_2_ to the GeSe monolayer, we compared the most stable configurations of CO_2_ that were adsorbed to the MoS_2_–GeSe monolayer. Our strategy was to calculate all typical adsorption structures and then the maximum adsorption energy would correspond to the most stable adsorption configuration.

*E*_b_ was determined using Equation (1), as follows:

*E*_b_ = *E*_MoS2–GeSe_ − *E*_GeSe_ − *E*_MoS2_ (1)

where *E*_MoS2–GeSe_, *E*_GeSe_ and *E*_MoS2_ represent the total energies of MoS_2_–GeSe, pure GeSe and MoS_2_ units, respectively.

*E*_ads_ was calculated using Equation (2), as follows:

*E*_ads_ = *E*_CO_2___/MoS2–GeSe_ − *E*_CO_2__ − *E*_MoS2–GeSe_ (2)

where *E*_CO_2___/MoS2–GeSe_, *E*_CO_2__ and *E*_MoS2–GeSe_ represent the energy of the total systems, CO_2_ and the separated systems, respectively.

The charge amount was obtained using the Milliken charge analysis and Δ*Q* was determined using Equation (3), as follows:

Δ*Q* = *Q*_a_ − *Q*_b_ (3)

where *Q*_a_ and *Q*_b_ represent the total charge of the gas molecules after and before adsorption, respectively.

## 3. Results

**Figure 1:**
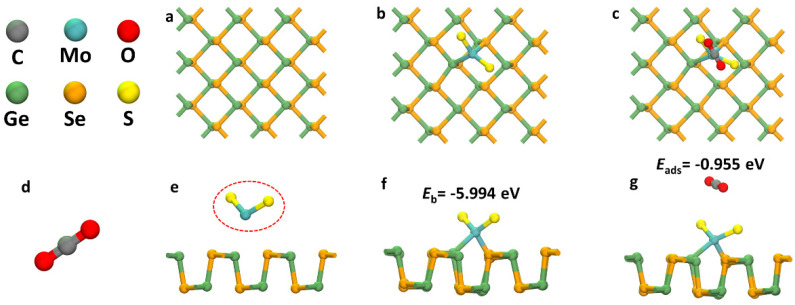
Geometric structures of the GeSe monolayer (**a**,**e**), the MoS_2_–GeSe monolayer (**b**,**f**), the CO_2_/MoS_2_–GeSe monolayer (**c**,**g**) and CO_2_ (**d**). The red circle is MoS_2_ unit.

As shown in Figure 1d, CO_2_ is a common compound in air that is composed of two oxygen atoms and a carbon atom, which are attached by a polar covalent bond. In Figure 1a,e, the ball–stick model structure of the pure GeSe monolayer is shown. GeSe monolayer are serrated black crystals that are similar to black phosphorus and belong to the orthogonal crystal system. After structural optimization, the Ge–Se bond lengths of the most stable GeSe monolayers were maintained at 2.641 and 2.544 Å. As shown in Figure 1b,f, the MoS_2_ units were stably adsorbed to the GeSe monolayer surface and the *E*_b_ value reached up to −5.994 eV, which indicated that a stable doping structure was formed with great thermal stability. In addition, the introduction of MoS_2_ units caused the Ge atoms in the GeSe monolayer to shift upward, which was produced by the strong interaction between the MoS_2_ units and the GeSe monolayer. As shown in Figure 1c,g, a stable structure with the maximum *E*_ads_ value was finally selected after the calculation of eight different sites. For the metal oxide materials, we found the four intrinsic structures of ZnO, SnO_2_, TiO_2_ and WO_3_ through a literature review and compared and explored the gas adsorption characteristics of CO_2_ to these common metal oxides. The specific parameters are shown in Table 1. It was obvious that the *E*_ads_ values of several metal oxides were much lower than those of the MoS_2_–GeSe monolayer (−0.955 eV) using DFT calculations, which indicated that the MoS_2_–GeSe monolayer had excellent response characteristics for CO_2_ detection. For pure ZnO, WO_3_ had some positive *E*_ads_ values, which indicated that the reaction could not be carried out spontaneously nor could that material be used as a sensor.

**Figure 2:**
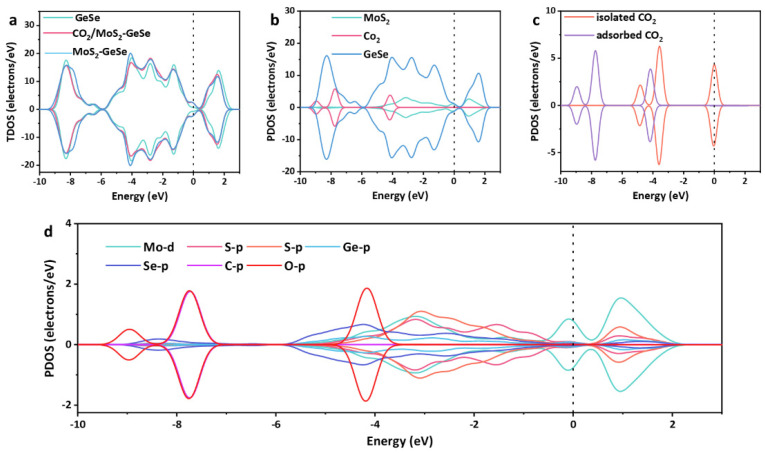
The TDOS and PDOS analyses of the CO_2_/MoS_2_–GeSe monolayer. The Fermi level is set at zero. (**a**) TDOS of CO_2_/MoS_2_–GeSe monolayer. (**b**) PDOS of CO_2_/MoS_2_–GeSe monolayer. (**c**) PDOS of two CO_2_. (**d**) PDOS of CO_2_/MoS_2_–GeSe monolayer with atoms.

As shown in Figure 2a, the overall TDOS of the MoS_2_–GeSe monolayer shifted to the right after doping with MoS_2_ units, especially near the Fermi level, which indicated that the conductivity of the GeSe monolayer decreased. In addition, a new peak appeared at −6 eV, which lays the foundation for the gas adsorption reaction. After the CO_2_ adsorption by the MoS_2_–GeSe monolayer, the peak amplitudes at −8.5 eV, −4 eV, −3 eV and 1.7 eV in the TDOS of the system increased slightly, which depended on the small regional state coating. This reflected the direct weak interactions between the two-dimensional materials and gases. We performed a PDOS analysis of the MoS_2_–GeSe monolayer decomposition (Figure 2b). Obviously, the adsorbed CO_2_, doping MoS_2_ units and modified GeSe had very obvious overlapping peaks at −8 eV and−4 eV, which indicated the existence of multi-electron orbital hybridization. After adsorption, we also found that the gas molecules did not produce new hybrid orbitals but rather shifted left overall. Therefore, we could conclude that the doped metal oxide had a tiny effect on the charge redistribution of the gas. In other words, the adsorbed CO_2_ could not redistribute the surface charge of the MoS_2_–GeSe monolayer or the electrical conductivity of the system, which revealed the corresponding response mechanisms of the gas sensor. As shown in Figure 2d, all elements showed high levels of hybridization and the orbitals were distributed between −10 and 2 eV, where Ge–p, Se–p, O–p and C–p orbitals contributed greatly to the charge recombination. The insertion of the p orbitals of O atoms into the MoS_2_ units caused electronic transitions in the gas molecules near the Fermi level, which formed new coupled states. In short, this indicated that there was an interaction between CO_2_, the MoS_2_ units and the GeSe monolayer; however, it was generally a weak interaction, which conformed to the application prospects of the MoS_2_–GeSe monolayer as a gas sensor.

**Figure 3:**
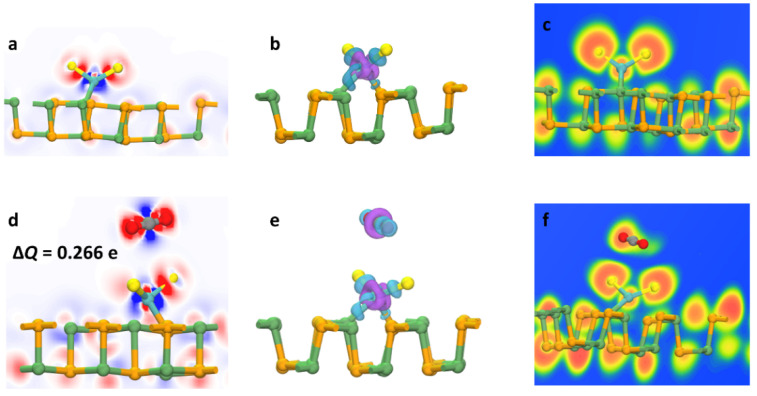
DCD (**a**), CDD (**b**) and ELF (**c**) on the MoS_2_–GeSe monolayer and DCD (**d**), CDD (**e**) and ELF (**f**) on the CO_2_/MoS_2_–GeSe monolayer.

In Figure 3a,d, the gain and loss of electron density are indicated by the blue and red regions, respectively. From the DCD distribution and Δ*Q* values, the gas molecules contributed 0.266 e to the material surface as electron donors and the electron density changed significantly between the different distribution regions. The electron density of the O atoms in CO_2_ increased greatly, while the region around the C atom lost electrons. The analysis indicated that the C atom of the CO_2_ molecule provided electrons not only to the two O atoms, but also to the O atom in the doped MoS_2_ units. It was evident from this transfer that the adsorption reaction was both exothermic and self-generated, which guaranteed stable and efficient adsorption effects. Figure 3b,e represents the redistribution of the charge density of CDD after adsorption. The adsorbed CO_2_ was charged positively overall and had purple areas around it, which indicated strong electron drainage. The separated charge build-up and exhaustion also confirmed the weak interaction between the gas and the adsorbent, with no new chemical bonds taking shape. As shown in Figure 3c,f, the ELF values were between −1 and 1, where −1 and 1 stand for very low electron density and completely limited electron density, respectively. It was found that stable O–Ge and Mo–Se bonds were formed between the MoS_2_ units and GeSe monolayer, which further confirmed the stability of the doping structure. In addition, there was no significant regional fusion between the CO_2_ and the MoS_2_–GeSe monolayer, which suggested that the adsorption reaction was physical adsorption and showed the uniqueness of the MoS_2_–GeSe monolayer as a gas sensor that allows for a faster response and recovery.

**Figure 4:**
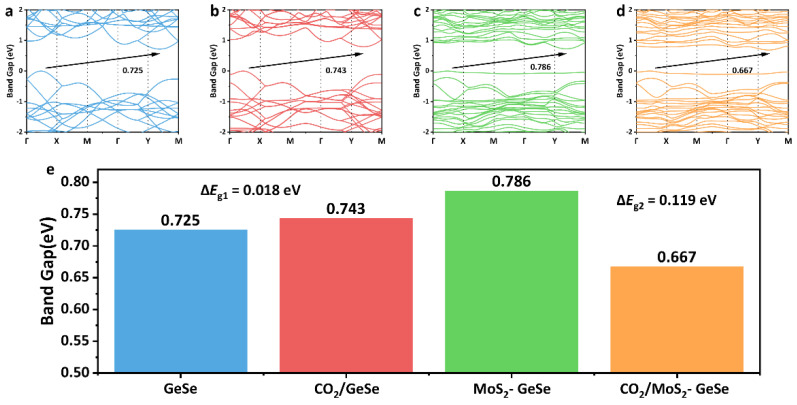
The *E*_g_ values of the pure GeSe monolayer (**a**), the CO_2_/GeSe monolayer (**b**), the MoS_2_–GeSe monolayer (**c**) and the CO_2_/MoS_2_–GeSe monolayer (**d**); the change trend of *E*_g_ (**e**).

The energy of electrons in solids cannot be continuously valued, but some discontinuous energy bands can be valued. To conduct electricity, there must be free electrons or holes. The energy of free electrons is called the conduction band and the energy of free holes is called the valence band. When the bound electrons become free electrons or holes, they must obtain enough energy from the valence band for the conduction band. The minimum energy is *E*_g_. In short, *E*_g_ refers to the energy transfer between the lowest level of the conduction band and the highest level of the valence band [54,55,56]:

σ ∝ A × exp (−Δ*E*_g_/2kT) (4)

where σ is the conductivity, k is the Boltzmann constant (1.38 × 10^−23^ J/K), A is a fixed parameter and T is the temperature. The change in *E*_g_ after an adsorption reaction can reflect the change in system conductivity and can further reflect the change in resistance value in practical applications. The *E*_g_ value of the MoS_2_–GeSe monolayer decreased after CO_2_ adsorption, which indicated that the conductivity of the GeSe-based monolayer increased and the resistance value decreased. As shown in Figure 4a,c, the doping with MoS_2_ units reduced the conductivity of the GeSe monolayer and increased the *E*_g_ value from 0.725 eV to 0.786 eV, which indicated that the MoS_2_–GeSe monolayer maintained the excellent semiconductor characteristics and gas response characteristics of pure GeSe. However, the *E*_g_ value of the pure GeSe monolayer after CO_2_ adsorption changed very little (from 0.725 eV to 0.753 eV), which indicated that CO_2_ had little effect on the electrical conductivity of the system (Figure 4b). In actual detection, it could be impossible to detect the resistance change of GeSe-based monolayer gas sensors using high precision instruments. The adsorption of CO_2_ reduced the *E*_g_ value of the MoS_2_–GeSe monolayer to 0.667 eV, which indicated that the difficulty of electron transition from the top of the valence band to the bottom of the conduction band was reduced (Figure 4d). This helped us to observe the resistance change that was caused by the gas concentration more clearly. In contrast to the pure GeSe monolayer, the MoS_2_–GeSe monolayer could be used as a gas sensor for CO_2_ detection.

**Figure 5:**
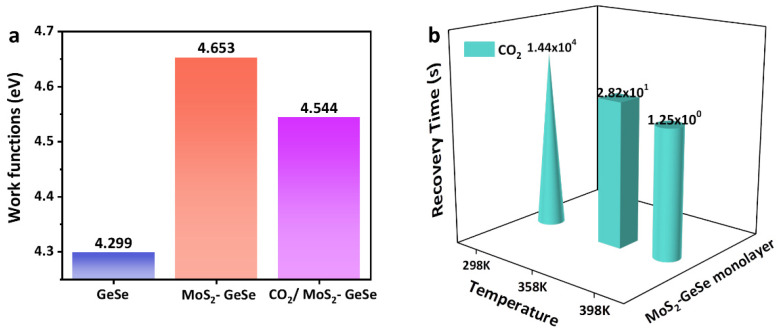
Change trend of WFs (**a**); recovery time at different temperatures (**b**).

WFs are a property of particular material surfaces, which indicate the minimum energy that is required for the surface to immediately move electrons to a point within a vacuum [57,58]. In gas interaction, this determines the aligned contact barrier between the gas molecules and specific surfaces. As shown in Figure 5a, the doping with MoS_2_ units increased the WFs of the GeSe monolayer to 4.653 eV, which hindered the electronic affinity of the system. This phenomenon further proved that the electronic transition of the GeSe monolayer was more difficult and that the chemical properties of the system were more stable after doping with MoS_2_ units. However, the introduction of CO_2_ reduced the WFs of the system to 4.544 eV, which indicated that after the CO_2_ was adsorbed, the difficulty of electron spillover from the vacuum reduced. This further guided the adsorption of the MoS_2_–GeSe monolayer to CO_2_. It also reflected the electrostatic attraction between the gas molecules and the adsorption system. It was this simultaneous attraction that led to the interaction between the CO_2_ and the MoS_2_–GeSe monolayer. In conclusion, gas adsorption could cause changes in WFs, which also proved the possibility of using a Kelvin probe microscope to detect WFs in the field of gas sensing. Zhang et al. also studied the process of gas adsorption using WFs [33,53,55,56,57,58,59,60,61]. Figure 5b presents the theoretical recovery time of CO_2_ at different temperatures that were calculated. The recovery times of sensing materials are closely related to the exposed ambient temperature. The calculation formula that we used was as follows [62,63]:

τ = v_0_^−1^ × exp (−*E*_ads_/kT) (5)

where k is the Boltzmann constant, τ is the recovery time and v_0_ is the attempt frequency (10^12^ s^−1^). The thermal environment offers more energy for the desorption process and fosters the further diffusion of molecules. At 298 K, the recovery time of CO_2_ was 14400 s, which caused the sensor to become unrecyclable. At 398 K, the desorption time of CO_2_ decreased to 1.25 s, which further illustrated the excellent performance of the MoS_2_–GeSe monolayer as a gas sensor. We inferred that 398 K could be used as the optimum temperature for the sensor, which would make it possible to apply the MoS_2_–GeSe monolayer in agriculture.

## 4. Conclusions

In this work, the sensing properties of an MoS_2_–GeSe monolayer for CO_2_ detection were systematically studied based on DFT. Compared to the pure GeSe monolayer, the MoS_2_–GeSe monolayer had a higher sensitivity to CO_2_ and the variation in *E*_g_ increased by 661% (0.119/0.018 × 100%). In practical applications, the MoS_2_–GeSe monolayer would have a greater change in resistance values as a chemical resistance sensor than during testing. The main parameters are shown in Table 2. The large *E*_b_ value (−5.994 eV) indicated that the MoS_2_ units and GeSe monolayer had excellent binding abilities. The thermal stability of the doped system was robust enough and the sensor could deal with changes in the ambient operating temperature. As can be seen from the DOS, the enriched orbital hybridization demonstrated the interaction between the CO_2_ and the active atomic groups. The ideal *E*_ads_ (−0.955 eV), Δ*Q* (0.266 e) and recovery time (1.25 s in 398 K) not only ensured the detection ability of the MoS_2_–GeSe monolayer as a sensor, but also ensured its excellent recyclability. This is an advantage that most two-dimensional material gas sensors do not have. Based on this theoretical investigation, we will proceed to test flexible patch gas sensors for the monitoring of crop growth.

## Figures and Tables

**Table 1:** The *E*_ads_ values of different materials.

2D Materials	Gas	*E*_ads_ (eV)	Ref.
ZnO	CO_2_	+0.01	[49]
ZnO	CO_2_	+0.44	[50]
SnO_2_	CO_2_	−0.663	[51]
TiO_2_	CO_2_	−0.17	[52]
ZrO_2_	CO_2_	−0.05	[52]
WO₃	CO_2_	0.22	[53]

**Table 2:** Main parameters.

	*E*_b_	*E*_ads_	Δ*Q*	WFs	Recovery Time
MoS_2_–GeSe Monolayer	−5.994 eV	/	/	4.653 eV	/
CO_2_/MoS_2_–GeSe Monolayer	/	−0.955 eV	0.266	4.544 eV	14,400 s (298 K) 28.2 s (358 K) 1.25 s (398 K)
